# Early Results of Acellular Dermal Matrix as an Interpositional Allograft Following Excision Arthroplasty of a Rheumatoid Forefoot: A Case Report

**DOI:** 10.5704/MOJ.2511.019

**Published:** 2025-11

**Authors:** D Huang, A Bhardwaj, WX Png, EWL Cher, IS Rikhraj

**Affiliations:** Department of Orthopaedic Surgery, Sengkang General Hospital, Singapore

**Keywords:** rheumatoid foot, spacer, arthrodesis, excision arthroplasty, allograft

## Abstract

This case report describes the use of a human acellular dermal matrix (ADM) as an interpositional allograft to stabilise and maintain the lesser metatarsophalangeal joint (MTPJ) pseudoarthrosis, and to prevent recurrent deformities, following arthrodesis of the first MTPJ and excision arthroplasty of the lesser metatarsals (MTs) for an elderly lady with severe rheumatoid forefoot deformities (RFDs). At six-month follow-up, the patient was recovering well with a clinically stable and radiographically well aligned foot, with no pain at the resection sites. She was also able to wear commercially available footwear.

## Introduction

Rheumatoid arthritis (RA) is an inflammatory polyarthropathy that can present with severe rheumatoid forefoot deformities (RFDs), most commonly hallux valgus (HV) and dislocation or destructive changes of the lesser metatarsophalangeal joints (MTPJs)^1^. These RFDs alter and disrupt the anatomy and biomechanical pressure distribution across the foot, causing more pain when weight bearing^1^. In the worst cases, forefoot reconstruction in the form of first MTPJ arthrodesis to stabilise the first ray and correction of lesser toe deformities with excision arthroplasty of the lesser metatarsal (MT) heads has been the surgical treatment of choice in relieving pain, correcting RFDs and achieving footwear comfort^1^.

Allografts can enhance surgical outcomes by avoiding donor site morbidity, reducing operative time, and effectively filling large defects in various tissues such as bone, ligaments, osteochondral lesions, and tendons^2^. Berlet *et al* reported on using a human acellular dermal matrix (ADM) in interpositional arthroplasty for hallux rigidus, replacing the damaged cartilage and bone^3^. This procedure showed encouraging results in their patient, with success reported at over a year after surgery.

We report the case of an elderly lady with painful and severe RFDs who underwent arthrodesis of the first MTPJ combined with interpositional implantation of human ADM allograft as a spacer after excising the lesser MT heads - to achieve a painless, stable and functional forefoot. She had previously undergone surgery to her forefoot six years earlier to address her RFD, which was unsuccessful. To the best of the authors’ knowledge, this is the first case report demonstrating the use of a biologically engineered spacer, specifically ADM, to treat severe RFDs.

## Case Report

An 83-year-old lady with diabetes mellitus and rheumatoid arthritis presented with a history of progressive, painful, right lesser MTPJ swelling and stiffness. She had difficulty in obtaining well-fitting commercially available footwear. Diagnostic radiographs of the involved foot revealed chronically dislocated lesser MTPJs with erosions involving the MT heads and proximal phalangeal (PP) bases, as well as a hallux valgus angle (HVA) of 37° and an intermetatarsal angle (IMA) of 14°. Bony osteopenia was also noted.

Treatment with nonsteroidal anti-inflammatory drugs (NSAIDs) did not significantly improve symptoms. She underwent scarf osteotomy, modified McBride procedure, lateral release and resection arthroplasty of the lesser MTPJs a year later.

**Fig. 1:**
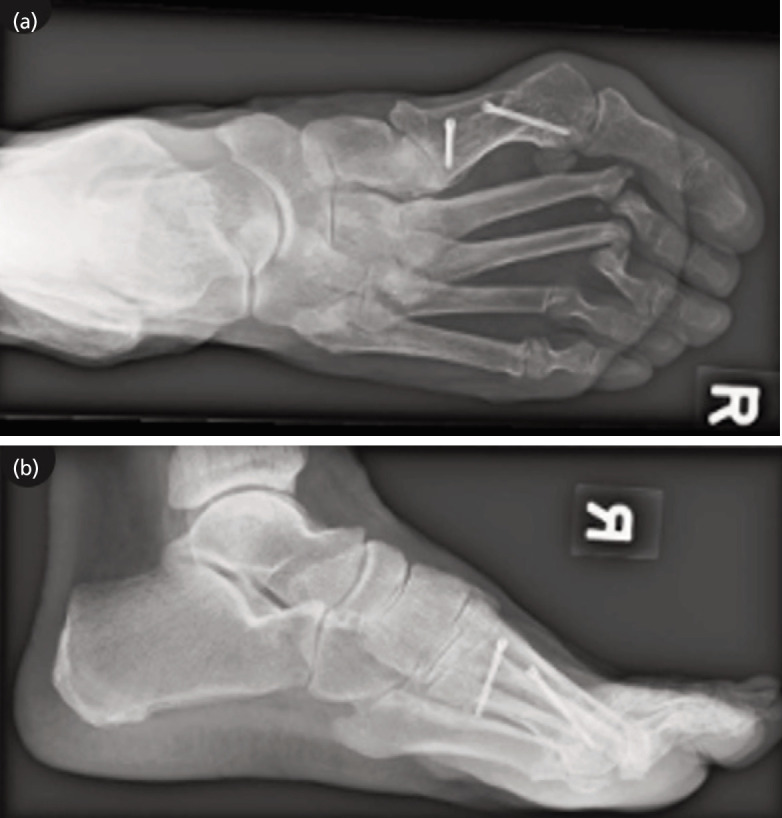
(a) AP and (b) lateral radiographs of the right foot showing a HV deformity with destruction of the MTPJs and degenerative changes of the first MTPJ and midfoot.

Six years after the primary surgery, the patient presented to our hospital with increasing pain scored as 8-9 on the 11-point visual analogue scale (VAS), as well as recurrent deformities in her right forefoot. Physical examination of the involved foot revealed tenderness at the plantar aspect of all metatarsophalangeal joints, with callosities present. The lesser toes were dorsiflexed and laterally deviated. Radiographs revealed lateral subluxation with pseudo-arthrosis at the previous resection sites of the lesser MTPJs together with osteoarthritic changes of the first MTPJ. The midfoot joints, although having osteoarthritic changes, were pain free (Fig. 1).

The patient underwent revision surgery in the form of first MTPJ arthrodesis and excision of the pseudoarthrosis sites with shortening of the lesser MTs to restore parabola of the forefoot.

The patient was positioned supine on the operating table after spinal anaesthesia had been administered. The approaches were a dorsomedial curvilinear incision over the first MTPJ, and two dorsal incisions between the second and third as well as the fourth and fifth MTs. Dissection was carried down to the first MTPJ capsule, where the MT was shortened and its articular surface excised. The bony surfaces were apposed and fixed with a plate and screws after the big toe was realigned, guided by an image intensifier.

**Fig. 2:**
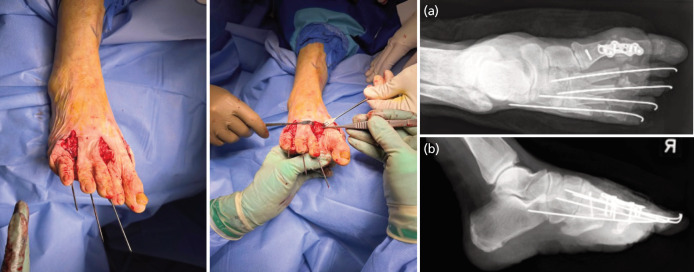
Intra-operative photographs of the right forefoot reconstruction, as well as post-operative, (a) AP and (b) lateral radiographs.

Subsequently, the lesser MTs were shortened relative to each other, through the dorsal approaches, to restore the parabola. Bone resection of between 4-10 millimetres was excised from the lesser MTs, following which the resulting gap at the distal ends of the MTs were allografted with interpositional ADM [AlloMend@AlloSource, Centennial, CO], secured with sutures through drill holes to the remnant MTs, and stabilised with K-wires (Fig. 2). Deliberate ranging of the lesser toes was done to test the stability of the grafts before K-wire fixation.

Copious amounts of sterile saline solution were used to flush the sites to remove any remaining bone fragments and debris. Layered closure was performed. A bulky compressive dressing was applied to the wound. The patient was placed in a forefoot offloading shoe with a prominent heel to allow partial weight bearing the foot, after the bulky dressing was removed on the second post-operative day.

**Fig. 3:**
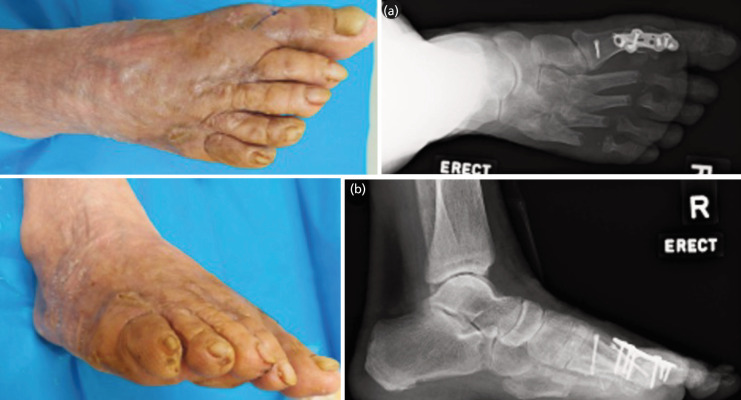
Latest clinical photographs of the right foot, as well as (a) AP and (b) lateral weight-bearing radiographs at the three-month follow-up.

She was kept partial weight bearing for eight weeks. Surgical stitches were removed at three weeks, when the wounds had healed. Two months after her surgery, the K-wires were removed and she was able to mobilise, full weight bearing, in commercially available sandals. Six months after the surgery, she was satisfied with no pain (VAS score 0) in her forefoot, which was clinically stable with good bony alignment radiographically (Fig. 3). HVA and IMA improved by 19° and 5° to become 18° and 9°, respectively on six-month follow-up radiograph. Regrettably, patient reported outcome measures such as the American Orthopaedic Foot and Ankle Society (AOFAS) score could not be recorded as she failed to attend the pre-operative diagnostic assessment.

## Discussion

Inflammation in the small joints of feet is characteristic of rheumatoid arthritis, and usually manifests in the early stages of the condition. The extent of structural damage and eventual joint failure can differ considerably between individuals, influenced by both the duration and severity of disease. Advanced disease with uncontrollable symptoms or deformities refractory to medical management becomes an indication for surgery^4^.

In our patient, the right forefoot deformity and pain recurred despite the previous surgery. A contemporary multidisciplinary approach including podiatry review for shoe modification and insoles all failed to alleviate her pain. Her seropositive RA had already been managed by a rheumatologist but found to be refractory to disease-modifying anti-rheumatic drugs (DMARDs). Indeed, despite significant progress made in the pharmacological treatment of RA with biologics, their efficacy in preventing the characteristic RFDs is not well quantified^4^.

Revision surgery consisting of arthrodesis of the 1st MTPJ and excision of the previous lesser excision arthroplasties together with ADM interposition arthroplasty was done, with the main indication for surgery being her forefoot metatarsalgia. The aims were to reproduce a stable pain-free forefoot, restore Lelièvre's parabola and normalise biomechanics during gait as much as possible, given the severe deformities of the forefoot. Our patient’s satisfaction, together with a pain-free clinically stable forefoot which is well-aligned radiographically, shows that the aims were met. Due to her diabetic neuropathy, she had a slower gait with shorter stride lengths along with reduced proprioception, though these were at baseline and not worse postoperatively.

Case reports have demonstrated the successful application of ADMs in the ankle, tarsometatarsal and first MTP joints, highlighting their versatility and reliability^4^. The literature suggests that this option provides a scaffold for host cell repopulation, revascularisation and ultimately the ingrowth potential to remodel into a robust arthrodial surface^5^. The novelty in this case is the use of a human dermal allograft for the interpositional arthroplasty and reconstruction of the lesser MTPJs.

Additionally, utilising an allograft eliminates the need to harvest tissue from the patient, reducing surgical complications. As this is a rather unique case, the authors have been unable to find any reports in the literature with regard to infection risk or rates associated with the use of this dermal allograft. Even being diabetic, the risk of infection in this patient was negligible as all wounds have healed primarily. She had been educated on foot and wound care as per our standard protocol for all diabetic patients. From the time of surgery till the last review, there had been no ulceration on the operated foot.

In the short term, our case report described a safe and successful reconstruction of severe lesser RFDs with adjunctive use of an interpositional spacer made of human dermal allograft. Six months after the surgery, the patient was able to fully resume her normal activities without any forefoot pain and wear commercially available footwear. Further longitudinal follow-up is required to determine if this reconstruction remains stable and pain free.
